# Prevalence of SARS-CoV-2 antibodies in healthy blood donors from the state of Tyrol, Austria, in summer 2020

**DOI:** 10.1007/s00508-021-01963-3

**Published:** 2021-10-26

**Authors:** Anita Siller, Gregor A. Wachter, Sabrina Neururer, Bernhard Pfeifer, Manfred Astl, Wegene Borena, Janine Kimpel, Sebastian Elmer, Franziska Spöck, Anja Vales, Annelies Mühlbacher, Manfred Gaber, Peter Willeit, Harald Schennach

**Affiliations:** 1 Central Institute for Blood Transfusion and Immunology, Tirol Kliniken GmbH, Anichstraße 35, 6020 Innsbruck, Austria; 2 Department of Clinical Epidemiology, Tyrolean Federal Institute for Integrated Care, Tirol Kliniken GmbH, Innsbruck, Austria; 3 Division for healthcare network and telehealth, UMIT—Private University for Health Sciences, Medical Informatics and Technology GmbH, Hall, Austria; 4 Institute for Virology, Department for Hygiene, Microbiology and Public Health, Medical University of Innsbruck, Innsbruck, Austria; 5 Blood donor service Tyrol of the Austrian Red Cross, Rum, Austria; 6 Clinical Epidemiology Team, Medical University of Innsbruck, Anichstraße 35, 6020 Innsbruck, Austria; 7 Department of Public Health and Primary Care, University of Cambridge, Cambridge, UK

**Keywords:** Seropositivity, Covid-19, Epidemiology, Seroprevalence, Cross-sectional studies

## Abstract

**Background:**

Seroepidemiological studies provide important insight into the spread of severe acute respiratory syndrome coronavirus 2 (SARS-CoV‑2) in our society. We aimed to determine seropositivity of SARS-CoV‑2 antibodies and its cross-sectional correlates in a large cohort of blood donors.

**Methods:**

In this observational cohort study, we tested healthy blood donors residing in Tyrol, Austria, for SARS-CoV‑2 antibodies using the Abbott SARS-CoV‑2 IgG chemiluminescent microparticle immunoassay. We estimated 95% confidence intervals (95% CI) of seroprevalences using bootstrapping and tested for differences by participant characteristics using logistic regression.

**Findings:**

Between 8 June and 4 September 2020, we screened 5345 healthy individuals at local blood donor sessions (mean age 42.7 years, SD 13.5 years, 46.7% female). Overall seroprevalence was 3.1% (95% CI 2.7–3.6%, 165 cases), which is 5.1-fold higher (95% CI 4.5–6.0%) than the case number identified by the health authorities in the state-wide testing program (0.6%; 4536 out of 757,634). Seroprevalence was higher in the district Landeck (16.6%, *P* < 0.001) and in individuals aged < 25 years (4.7%, *P* = 0.043), but did not differ by gender, blood types, or medication intake. The odds ratio for seropositivity was 2.51 for participants who had travelled to Ischgl (1.49–4.21, *P* = 0.001), 1.39 who had travelled to other federal states (1.00–1.93, *P* = 0.052), and 2.41 who had travelled abroad (1.61–3.63, *P* < 0.001). Compared to participants who had a suspected/confirmed SARS-CoV‑2 infection but were seronegative, seropositive participants more frequently reported loss of smell (odds ratio = 2.49, 1.32–4.68, *P* = 0.005) and taste (odds ratio = 2.76, 1.54–4.92, *P* = 0.001).

**Conclusion:**

In summer 2020, SARS-CoV‑2 seroprevalence in Tyrolean blood donors was 3.1%. Our study revealed regional variation and associations with young age, travel history and specific symptoms.

## Introduction

Coronavirus disease 2019 (COVID-19) caused by the severe acute respiratory syndrome coronavirus 2 (SARS-CoV-2) was first detected in December 2019 in Wuhan, province Hubei, China [1] and spread rapidly worldwide including Europe [2]. Typical symptoms of COVID-19 are fever, persistent cough, shortness of breath [1] and many patients also experience olfactory and gustatory dysfunctions [3, 4]. Symptoms are present at different ages, with varying frequency and severity [1, 5, 6]. While the majority of cases show mild or even no symptoms, a proportion of cases often with comorbidities require intensive care unit (ICU) admission and/or prolonged mechanical ventilation [1, 3].

In Austria, the first two confirmed COVID-19 cases, an Italian couple entering from Lombardy, Italy, were diagnosed in Innsbruck, Tyrol on 25 February 2020 [7]. Following an outbreak in the skiing area of Ischgl, Tyrol, [3, 7] and a steep increase in documented SARS-CoV-2 cases in the whole federal state in the first half of March 2020, comprehensive preventive measures were put in place, including quarantine of all municipalities in Tyrol. The number of SARS-CoV-2 cases plateaued in the end of March at a 7-day incidence rate of 150–160 cases per 100,000 inhabitants and subsequently declined with very low rates from May until July 2020 (< 5 cases per 100,000 inhabitants), before steadily rising again thereafter [8]. Altogether, from March to September 2020, health authorities identified 4536 cases, corresponding to 0.6% of the total population in the state of Tyrol (*n* = 757,634); however, given limited testing capacities during the first epidemic peak, the focus on symptomatic cases or on those with a travel history, and challenges in contact tracing, it is likely that this number is an underestimate of the true burden of disease in the Tyrolean population. For instance, in the skiing area of Ischgl, 42.4% of the population were seropositive, but only one fifth of those had previously been identified as SARS-CoV-2 cases [3].

To address this uncertainty, we conducted a seroprevalence study in 5345 healthy blood donors in Tyrol. Our aims were threefold. First, to quantify the prevalence of SARS-CoV‑2 IgG/IgM antibodies in this study population. Second, to estimate the proportion of cases captured by the health authorities through the state-wide testing program. Third, to identify factors associated with high or lower seroprevalence, thereby determining whether certain population subgroups were disproportionally affected by SARS-CoV‑2 infection.

## Participants, material and methods

### Sampling and recruitment

Between 8 June and 4 September 2020, we recruited blood donors at local blood donation events to take part in this epidemiologic study. To be eligible for participation, donors had to (i) meet the general requirements for a blood donation; (ii) be living in Tyrol; (iii) be in a healthy state (for instance, free of malignant diseases, autoimmune diseases or infectious diseases); and (iv) be asymptomatic for at least 28 days in case they had had a PCR-confirmed or suspected SARS-CoV‑2 infection. Out of 7244 potentially eligible individuals, a total of 5345 individuals took part in the study, corresponding to a participation rate of 73.8%. Blood donors of a broad age range (18–71 years) and of both sexes were included. The present study was approved by the local ethics committee of the Medical University Innsbruck (1134/2020) and participants provided written informed consent.

### Study questionnaire and telephone survey

All participants of the study were asked to complete a 2-page questionnaire at the time of blood donation. The questionnaire included information on age, sex, place of residence, blood group, and intake of medication during the preceding 4 weeks. We also gathered information on whether participants had travelled abroad in the 6 months preceding the blood donation, or whether they had visited any COVID-19 hotspot areas in Austria or other Austrian federal states since 1 December 2019. Furthermore, we asked participants to report whether (and if yes, when) they thought they had gone through a SARS-CoV‑2 infection, whether infection was confirmed with PCR, diagnosed by a medical doctor, or suspected by the affected individual. The term “suspected infection” is used if people thought to have gone through a SARS-CoV‑2 infection because of the symptoms they had or if they were in contact with a confirmed SARS-CoV‑2 case.

We also conducted a telephone survey in a subset of study participants in order to compare the spectrum and duration of symptoms. Symptoms queried included cough, sore throat, limb pain, shortness of breath, dyspnea, headache, vomiting/nausea, diarrhea, anosmia and/or ageusia. Other questions recorded were need for further treatment, such as hospitalization, ICU treatment or if a medical doctor was consulted due to severity of symptoms. Finally, the outcome of prior antibody and/or PCR testing was addressed.

### Sample collection and laboratory analysis

Serum samples of blood donors were screened for SARS-CoV‑2 IgG antibodies with the Abbott Alinity i instrument using the Abbott SARS-CoV‑2 IgG assay (Abbott Ireland, Sligo, Ireland), a high-throughput chemiluminescent microparticle immunoassay (CMIA) used for qualitative detection of IgG antibodies against SARS-CoV‑2 nucleocapsid protein, according to the manufacturer’s instructions. The performance (specificity/sensitivity) of this assay was validated previously [9, 10]. According to the manufacturer’s information, a sensitivity of 100% (95% confidence interval, CI, 95.89–100.0%) in samples analyzed 14 days after symptom onset and a specificity of 99.63% (95% CI 99.05–99.90) [11] could be expected. When blood donors were tested positive in this first assay, the WANTAI SARS-CoV‑2 Ab enzyme-linked immunosorbent assay (ELISA, Beijing Wantai Biological Pharmacy Enterprise, Beijing, China), detecting total SARS-CoV‑2 antibodies (IgG and IgM) directed against the SARS-CoV‑2 spike protein, was performed according to manufacturer’s instructions. When both tests were positive, samples were additionally analyzed using the WANTAI SARS-CoV‑2 IgM ELISA (Beijing Wantai Biological Pharmacy Enterprise) according to manufacturer’s instructions and an in-house neutralization assay. The neutralization test was based on a replication defective vesicular stomatitis virus (VSV) vector, which is pseudotyped with a C-terminally truncated spike protein of SARS-CoV‑2 (VSV‑S [12]) and encodes green flourescent protein (GFP) as marker gene. Briefly, vectors were pre-incubated with fourfold dilutions of heat-inactivated donor samples for one hour and subsequently HEK293T cells stably overexpressing angiotensin I converting enzyme 2 were infected with these mixtures. After 16 h, infected cells, i.e. GFP positive cells, were counted using an ImmunoSpot S6 Ultra‑V reader and FluoroSpot software (CTL Europe, Bonn, Germany). The antibody dilution, which resulted in > 50% reduction of the number of GFP positive cells as compared to the virus only control was defined as neutralizing titer. Neutralization titers ≤ 1:4 were regarded as negative.

### Statistical analysis

Data are given as absolute frequencies (*n*) and percentages (%). For comparisons of seropositivity between different groups, odds ratios (OR) and 95% confidence intervals (CI) were calculated using binary logistic regressions. All statistical analyses were performed using SPSS 26 (IBM Corp. Released 2019. IBM SPSS Statistics for Windows, Version 26.0. Armonk, NY, USA) or Stata 16 (StataCorp. 2019, Stata Statistical Software: Release 16. College Station, TX, USA). *P* values < 0.05 were considered statistically significant.

## Results

**Fig. 1:**
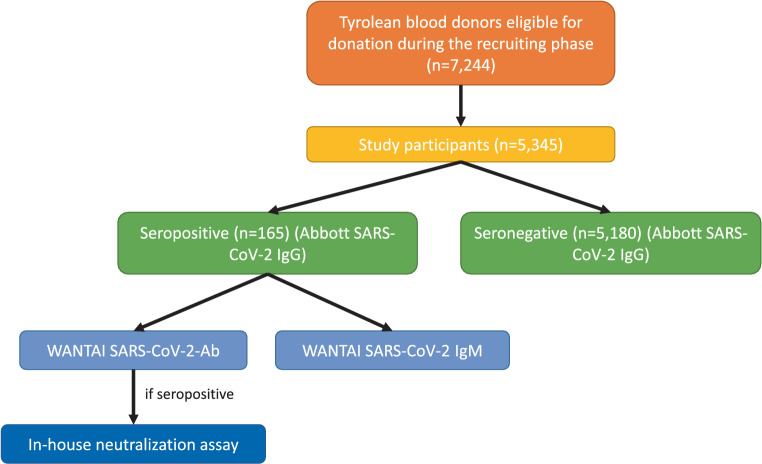
Testing procedure with different assays

In the present study, we investigated prevalence of SARS-CoV‑2 IgG antibodies directed against the nucleocapsid protein of SARS-CoV‑2, using the Abbott SARS-CoV‑2 IgG CMIA, in a cohort of 5345 healthy Tyrolean blood donors (Fig. 1). All blood donors were required to be asymptomatic at the time of sample collection. Median age of participants was 47 years (range: 18–71 years); 2497 were female (46.7%) and 2848 were male (53.3%).

### Overall seroprevalence of SARS-CoV-2 antibodies

Of the 5345 individuals screened, 165 were seropositive for SARS-CoV‑2 IgG antibodies (Fig. 1). This corresponds to a seroprevalence of 3.1% (95% CI 2.6–3.6%), which is 5.1-fold higher (95% CI 4.5–6.0%) than the case number identified by the health authorities in the state-wide testing program (0.6%; 4536 out of 757,634) [8].

We then conducted detailed additional laboratory analysis on the 165 positive samples (Fig. 1). First, we retested the positive samples using the WANTAI SARS-CoV-2-Ab ELISA, which detects both IgM and IgG antibodies directed against the spike protein. The majority of samples were again positive (134 out of 165; 81.2%), whereas one sample was borderline (0.6%) and 28 were negative (17.0%). Of the previously positive samples, two (1.2%) were not retested with the second assay due to technical problems. If only double-positive individuals (spike and nucleocapsid antibodies) were considered as positive, overall seroprevalence would be 2.5% (95% CI 2.1–3.0%).

Second, we retested the 165 positive samples using the WANTAI SARS-CoV-2-IgM ELISA in order to resolve the percentage of blood donors harboring IgM antibodies directed against the spike protein of SARS-CoV‑2. This test showed that 65 samples were IgM antibody positive (39.4%), 10 were borderline (6.1%), and 82 were IgM antibody negative (49.7%). Due to technical problems 8 (4.8%) of the double-positive samples could not be analyzed for IgM antibodies.

Finally, in the 134 samples that were positive with the Abbott SARS-CoV‑2 IgG CMIA and the WANTAI SARS-CoV-2-Ab ELISA, we performed an in-house neutralization assay to investigate if the antibodies are capable of neutralizing SARS-CoV‑2. All the samples we tested in the neutralization assay showed a titer of at least 1:16 or higher. In total, 48 (35.8%) showed a neutralization titer of 1:16, 51 (38.1%) of 1:64, 28 (20.9%) of 1:256, 3 (2.2%) of ≥ 1:1024, and 4 were not tested.

### Seroprevalence by age, sex and blood type

**Fig. 2:**
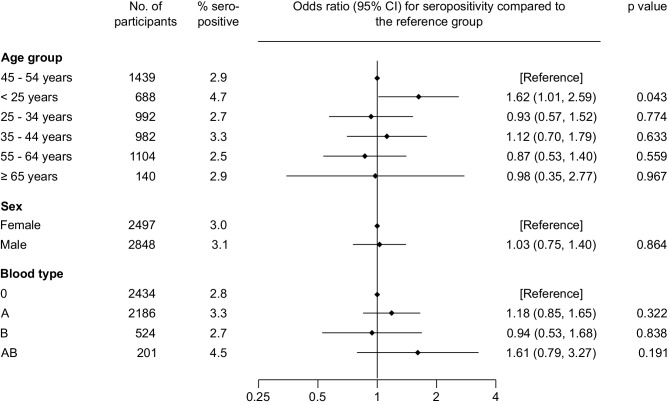
Seroprevalence of SARS-CoV‑2 IgG antibodies in the Tyrolean blood donor cohort according to age groups, sex and blood types

Fig. 2 shows SARS-CoV‑2 IgG seroprevalences and odds ratios by age, sex and blood type. Among 2497 female and 2848 male blood donors, 76 (3.0%) and 89 (3.1%) were tested positive for SARS-CoV‑2 IgG antibodies, respectively. Seropositivity was not significantly different (OR = 1.03, 95% CI 0.75–1.40, *P* = 0.864) between female and male individuals. When we analyzed different age groups, the odds of being IgG seropositive was 1.62-fold higher in participants aged < 25 years than in the reference group of participants aged 45–54 years (95% CI 1.01–2.59; *P* = 0.043; 4.7% seropositive vs. 2.9% seropositive), but we observed no significant differences in seropositivity in the other age groups of 25–34 years, 35–44 years, 55–64 years and ≥ 65 years, compared to reference group. Seroprevalence was also similar when we grouped participants according to their blood type (all *P* > 0.05).

### Seroprevalence throughout the nine districts of Tyrol

**Fig. 3:**
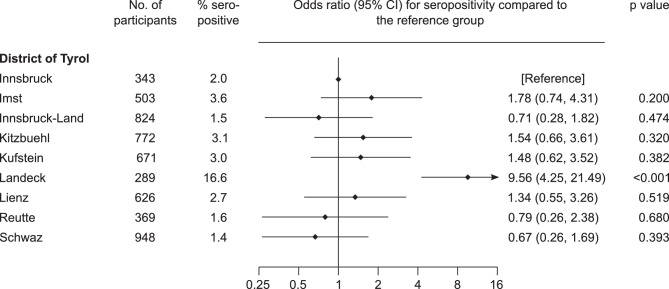
Comparison of seroprevalence of SARS-CoV‑2 IgG antibodies in the Tyrolean blood donor cohort across the nine districts of Tyrol

We analyzed SARS-CoV‑2 IgG seroprevalence among blood donors according to their places of residence grouped by the nine districts of Tyrol (Fig. 3). While the odds of seropositivity did not differ for most districts, we observed a highly elevated seroprevalence in the district of Landeck (16.6%). When compared to the district of Innsbruck (seroprevalence 2.0%), the odds ratio for seropositivity was 9.56 (95% CI 4.25–21.49, *P* < 0.001). In a secondary analysis restricted to the hotspot region Ischgl (which lies in the district of Landeck), 30 out of 91 blood donors were seropositive, corresponding to a seroprevalence of 33.0%.

### Seroprevalence according to self-reported travel history

**Fig. 4:**
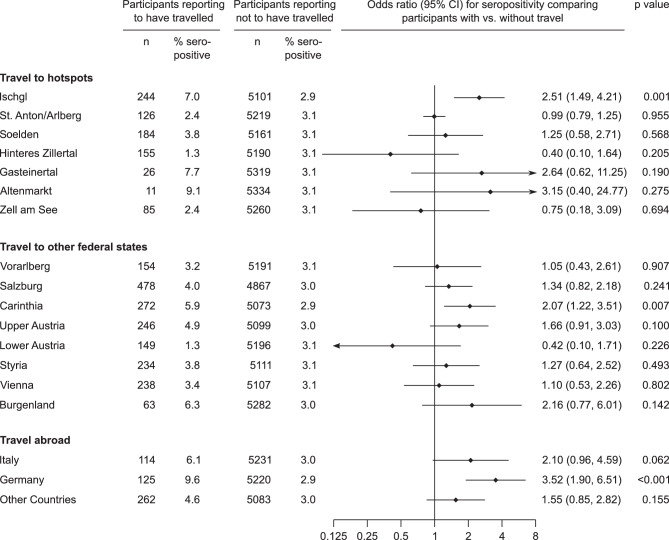
Seroprevalence of SARS-CoV‑2 IgG antibodies in the Tyrolean blood donor cohort according to self-reported travel history. The analysis of travel to hotspots excluded participants already living in the respective regions. The region “Hinteres Zillertal” included the municipalities Finkenberg, Tux, Schwendau, Mayrhofen, Brandenberg, Ramsau, Heinzenberg, and Hippach. The region St. Anton/Arlberg included the municipalities St. Anton, Pettneu, Strengen and Flirsch. The questionnaire addressed travel history to other federal states starting from 1 December 2019, whereas travels to countries abroad were addressed for the preceding six months

We compared seroprevalence between participants who reported to have travelled and participants who reported to have not travelled, distinguishing between three types of travel destinations (all shown in Fig. 4). First, we assessed seroprevalence by self-reported travel to certain hotspot regions since 1 December 2019, excluding participants that permanently lived in these hotspot regions. In this analysis, we found a higher seroprevalence among the 244 participants reporting to have visited Ischgl (7.0%) than the 5101 participants who did not (2.9%), corresponding to an odds ratio for seropositivity of 2.51 (95% CI 1.49–4.21, *P* = 0.001). The odds ratios for travel to any of the other hotspot regions were not significant, although statistical power was limited for some due to small group sizes (e.g. Altenmarkt, Gasteinertal).

**Fig. 5:**
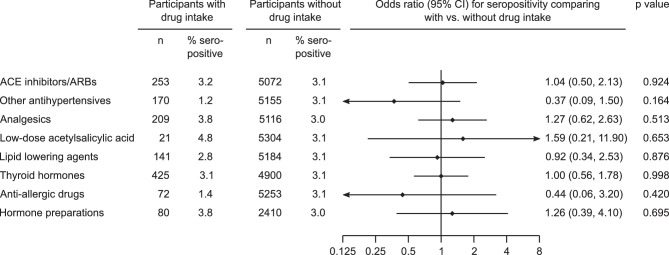
Seroprevalence of SARS-CoV‑2 IgG antibodies in the Tyrolean blood donor cohort according to self-reported medication intake. *ACE* angiotensin converting enzyme, *ARBs* angiotensin receptor blockers

Second, we analyzed whether seroprevalence differed by self-reported travel to other Austrian federal states since 1 December 2019. Seroprevalence was 3.8% among the 1429 participants who travelled to other Austrian federal states and 2.8% among the 3916 participants who did not, corresponding to an odds ratio for seropositivity of 1.39 (95% CI 1.00–1.93, *P* = 0.052). Results for the individual federal states of Austria are shown in Fig. 4. While odds to be seropositive were elevated among participants with a recent travel to Carinthia (OR: 2.07, 95% CI 1.22–3.51, *P* < 0.007), they did not differ significantly by travel history to other federal states.

Third, we evaluated whether participants travelled abroad in the 6 months preceding the blood donation. Seroprevalence was 6.4% among the 467 participants reported to have travelled abroad and 2.8% among the 4878 participants who did not, corresponding to an odds ratio for seropositivity of 2.41 (95% CI 1.61–3.63, *P* < 0.001). The most common travel destination was Germany, associated with an odds ratio of 3.55 (95% CI 1.92–6.57, *P* < 0.001) of being seropositive (Fig. 4).

### Seroprevalence according to self-reported medication intake

To evaluate if the intake of certain medications was associated with higher or lower prevalence, we assessed medication intake categorized by predefined different drug classes. When we compared seroprevalence according to intake of medication, we observed no significant differences by any of the drug classes angiotensin converting enzyme inhibitors/angiotensin receptor blockers, other antihypertensive drugs, analgesics, low-dose acetylsalicylic acid, lipid lowering agents, thyroid hormones, anti-allergic drugs and hormone preparations (i.e. hormonal contraception, postmenopausal hormone replacement therapy, all *P* > 0.05) (Fig. 4).

### Assessment of self-reported symptoms in a subset of the study population

**Fig. 6:**
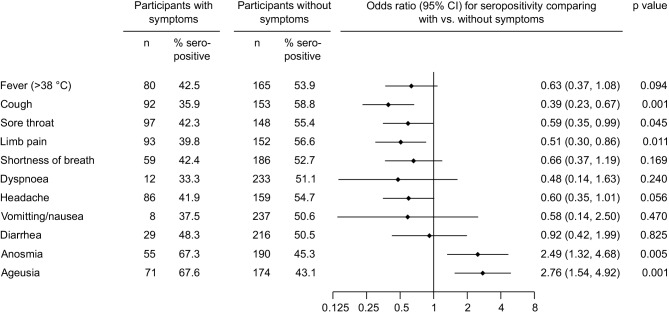
Seroprevalence of SARS-CoV‑2 IgG antibodies in the Tyrolean blood donor cohort according to self-reported symptoms

We conducted a telephone survey to assess self-reported symptoms among 123 participants who were seropositive and 122 participants who were seronegative and had suspected having had an infection or had a laboratory confirmed SARS-CoV‑2 infection in the past. The telephone survey covered the symptoms fever (> 38 °C), cough, sore throat, limb pain, shortness of breath, dyspnea, headache, vomiting/nausea, diarrhea, anosmia and ageusia. Of them, anosmia (OR = 2.49, 95% CI 1.32–4.68, *P* = 0.005) and ageusia (OR = 2.76, 95% CI 1.54–4.92, *P* = 0.001) were linked to higher odds of being seropositive, whereas cough (OR = 0.39, 95% CI 0.23–0.67, *P* = 0.001) and limb pain (OR = 0.51, 95% CI 0.30–0.86, *P* = 0.011) were linked to lower odds of being seropositive (Fig. 6). Out of the 123 seropositive participants, 30 reported none of the aforementioned symptoms (24.4%).

## Discussion

The present study reports on the seroprevalence of SARS-CoV‑2 antibodies in 5345 healthy individuals recruited at local blood donor sessions in the federal state of Tyrol, Austria. Our study shows that, in summer 2020, seroprevalence was 3.1% and therefore approximately five times higher than expected based on the number of cases identified through the state-wide testing program in place at that time. A comparable gap in the detection of SARS-CoV-2 cases at the start of the pandemic in spring 2020 has been previously shown by a study conducted in Vienna [13]. In a series of cross-sectional association analyses, we furthermore demonstrate regional variation, with a substantially higher seroprevalence in the district of Landeck including Ischgl, which was the setting of a major outbreak and a separate seroprevalence study [3]. Finally, we also detected a higher seroprevalence in the youngest participants, in those with a travel history and those reporting the symptoms loss of smell and loss of taste.

Our study is the first to quantify seroprevalence of SARS-CoV‑2 antibodies in the whole federal state of Tyrol, Austria, estimating it at 3.1%. A previous study conducted among 1473 inhabitants of the Austrian ski resort Ischgl has shown a seroprevalence of 42.4% in the end of April 2020 [3] and an absolute reduction of 6.7% among 801 individuals with a repeat measurement after a 6-month follow-up [14]. In line, we also show a higher seroprevalence of 16.6% in the whole district of Landeck and 33.0% among blood donors living in Ischgl, whereas seroprevalence in other districts of Tyrol was substantially lower. Finally, a separate nationwide seroepidemiological study conducted mid-November involved 2229 individuals aged ≥ 16 years and showed a seroprevalence of 4.7% (95% CI 3.8–5.6%) across the whole of Austria, but did not afford the statistical power to allow determination of state-specific prevalence estimates [15]. Although blood donors are a healthy subgroup of the overall population, we are able to provide a unique insight into the proportion of the population that had been infected with SARS-CoV‑2 in all Tyrolean districts and therefore reveal the “true” burden of disease at a population-level. We furthermore conducted subsidiary laboratory analyses with largely confirmatory findings, including presence of both anti-spike IgG and IgM antibodies as well as neutralization titers of 1:16 or higher; however, the majority of our blood donors only had rather low or intermediate neutralization titers (1:16 or 1:64) whereas only few had titers of 1:256 or higher. This could potentially correlate with the generally mild courses of disease in our blood donors. Finally, our results are in line with reports from blood donors of four other Austrian federal states [16], of the Lombardy region in Italy [17], the San Francisco Bay area in the USA [18] and recent findings from the Mount Sinai Health System in New York City [19].

Our study also assessed the association of self-reported symptoms with seropositivity. In general, blood donors tend to be of good general health (i.e. free of chronic diseases such as malignoma, diabetes, or autoimmune diseases, and younger than 70 years old) and, consequently, blood donors may have a lower risk for severe COVID-19 disease courses. In our study, self-reported symptoms were generally mild, with about one quarter of the seropositive donors being completely asymptomatic. Our study however shows that—among a comprehensive list of symptoms we investigated—loss of taste and/or smell were significantly more often reported by participants who were IgG positive than those who were IgG negative, thereby endorsing previous reports that these symptoms may be highly indicative for past SARS-CoV‑2 infection [3, 4, 13].

When we analyzed seroprevalence across prespecified age groups, we observed a higher percentage of seropositivity in younger individuals (< 25 years). This could be due to the higher number of contacts among the young participants, but also due to the higher mobility in this age group as we could show that travel history to some places was also associated with a higher seroprevalence. Concerning the presence of SARS-CoV‑2 antibodies in male vs. female blood donors, we did not observe a statistically significant difference, which confirms other previous studies [20]. We also did not observe an association of higher or lower seroprevalence in blood donors with distinct blood types in our cohort, although it was previously reported that blood type O could be a protective factor for COVID-19 [21]. Furthermore, we were also interested in potential associations of medication intake with seroprevalence, as especially ACE-Is and ARBs were discussed in this context previously [22] but did not observe significant differences when comparing participants with vs. without medication intake.

Our study has strengths and limitations. The strength of the present study is that our dataset spans throughout all districts of Tyrol and is adequately sized, thereby allowing to explore differences by region and several other characteristics. Nevertheless, a limitation of this study is that it excluded individuals with specific comorbidities which are known to influence severity and disease progression of COVID-19. This limitation likely has little impact on the overall seroprevalence but does affect our analysis on the frequency of self-reported symptoms. Even though our study included 5345 blood donors in total, some of the subgroup analyses need to be interpreted with caution due to low sample numbers and warrant further investigation; however, knowing that not everybody develops detectable antibodies after a confirmed COVID-19 infection [23] and that SARS-CoV‑2 antibodies can wane within a short period of time after the infection [24, 25], we assume that even more people went through a COVID-19 infection as can be estimated by the present study.

In summary, SARS-CoV-2 seroprevalence was 3.1% in summer 2020 among Tyrolean blood donors. Our study underscores the suitability of blood donors as an epidemiologic sentinel surveillance system in order to identify local outbreaks and monitor diseases, as blood donors are easily available and representative, at least for the generally healthy subgroup of our population [16, 26].
